# Standardisation of information submitted to an endpoint committee for cause of death assignment in a cancer screening trial – lessons learnt from CAP (Cluster randomised triAl of PSA testing for Prostate cancer)

**DOI:** 10.1186/1471-2288-15-6

**Published:** 2015-01-23

**Authors:** Naomi J Williams, Elizabeth M Hill, Siaw Yein Ng, Richard M Martin, Chris Metcalfe, Jenny L Donovan, Simon Evans, Laura J Hughes, Charlotte F Davies, Freddie C Hamdy, David E Neal, Emma L Turner, CAP Cause of Death Committee**

**Affiliations:** School of Social and Community Medicine, University of Bristol, based at Royal Hallamshire Hospital, Sheffield, S10 2JF UK; School of Social and Community Medicine, University of Bristol, Canynge Hall, Bristol, BS8 2PS UK; School of Social and Community Medicine, University of Bristol, based at Freeman Hospital, High Heaton, Newcastle-upon-Tyne, NE7 7DN UK; Royal United Hospital, Bath, BA1 3NG UK; Department of Oncology, University of Cambridge, Addenbrooke’s Hospital, Cambridge, CB2 0QQ UK; Nuffield Department of Surgical Sciences, University of Oxford, John Radcliffe Hospital, Oxford, OX3 9DU UK

**Keywords:** Underlying cause of death, Verification, Bias, Prostate cancer, Blinding, Standardisation of information, Trial arm, Endpoint review, Outcome assessors

## Abstract

**Background:**

In cancer screening trials where the primary outcome is target cancer-specific mortality, the unbiased determination of underlying cause of death (UCD) is crucial. To minimise bias, the UCD should be independently verified by expert reviewers, blinded to death certificate data and trial arm. We investigated whether standardising the information submitted for UCD assignment in a population-based randomised controlled trial of prostate-specific antigen (PSA) testing for prostate cancer reduced the reviewers’ ability to correctly guess the trial arm.

**Methods:**

Over 550 General Practitioner (GP) practices (>415,000 men aged 50–69 years) were cluster-randomised to PSA testing (intervention arm) or the National Health Service (NHS) prostate cancer risk management programme (control arm) between 2001 and 2007. Assignment of UCD was by independent reviews of researcher-written clinical vignettes that masked trial arm and death certificate information. A period of time after the process began (the initial phase), we analysed whether the reviewers could correctly identify trial arm from the vignettes, and the reasons for their choice. This feedback led to further standardisation of information (second phase), after which we re-assessed the extent of correct identification of trial arm.

**Results:**

1099 assessments of 509 vignettes were completed by January 2014. In the initial phase (n = 510 assessments), reviewers were unsure of trial arm in 33% of intervention and 30% of control arm assessments and were influenced by symptoms at diagnosis, PSA test result and study-specific criteria. In the second phase (n = 589), the respective proportions of uncertainty were 45% and 48%. The percentage of cases whereby reviewers were unable to determine the trial arm was greater following the standardisation of information provided in the vignettes. The chances of a correct guess and an incorrect guess were equalised in each arm, following further standardisation.

**Conclusions:**

It is possible to mask trial arm from cause of death reviewers, by using their feedback to standardise the information submitted to them.

**Trial registration:**

ISRCTN92187251

**Electronic supplementary material:**

The online version of this article (doi:10.1186/1471-2288-15-6) contains supplementary material, which is available to authorized users.

## Background

In trials of cancer screening, where the primary outcome is target cancer-specific mortality, the accurate determination of cause of death is crucial. The use of an independent panel of experts to assign underlying cause of death (UCD) following a review of medical notes is usually regarded, with the exception of autopsy, as the gold standard [1–4] and in most countries is preferable to the use of death certificates alone, where doubt may exist about the overall quality of cause of death certification [5–7]. This is especially true in trials where the population is elderly with multiple, competing co-morbidities or malignancies [8–10]. In these circumstances a degree of misclassification of cause of death is inevitable, but if this is unrelated to trial arm (non-differential misclassification) then the effect of screening will be modestly underestimated at worst [11]. However, substantial bias such that the effect of screening is over or underestimated may arise if misclassification is worse in one trial arm than the other (differential misclassification). Differential misclassification may be avoided by blinding panel experts to the trial arm a participant was in.

In cancer screening trials differential misclassification may arise from two well-known sources of potential death certificate bias. First, ‘sticking-diagnosis’ or attribution bias, which arises because more target cancers are diagnosed in the intervention arm and therefore deaths are more likely to be attributed to that cancer compared to the control arm [1, 12]. Secondly, deaths due to the screening process itself which are not traced back to screening but are certified as due to other causes will lead to an overestimation of the beneficial effects of screening [1, 12]. Such ‘slippery-linkage’ bias might arise from complications during the diagnostic process or following specific therapeutic interventions for screen-detected disease (such as complications following surgery for the screen-detected cancer). The use of all-cause mortality as an alternative endpoint avoids problems of attribution bias and includes unattributed deaths due to screening, but requires very large numbers of trial participants contributing many person-years of observation. For this reason, most cancer screening trials use target cancer-specific mortality as the primary outcome, while seeking to minimise the effect of these biases through the review of medical notes and assignment of UCD by an endpoint committee blind to allocation [13–15].

The accurate assignment of UCD by an endpoint committee requires identical methods of data collection across trial arms and masking of reviewers to both the allocated trial arm and the screening status of individuals [1]. A major criticism of the early breast cancer screening trials was that endpoint committee reviewers were fully aware of which women had been screened [16]. In cancer screening trials it is a challenge to conceal the trial arm from cause of death reviewers without compromising the accurate verification of UCD. Nevertheless, to optimise masking of trial arm, one guiding principle should be that the type of information presented to reviewers must be standardised [1].

This paper investigated whether the standardisation of information submitted to an endpoint committee for UCD ascertainment in the ongoing Cluster randomised triAl of PSA testing for Prostate cancer (CAP) (ISRCTN92187251) successfully reduced the ability of reviewers in correctly guessing the trial arm, thereby minimising any potential biases arising from such guesses. Trained researchers abstracted clinical information from medical records and wrote short structured vignettes that were independently reviewed by a team of clinicians, each of whom separately assigned an UCD. To reduce bias in the verification process, vignettes were carefully worded and followed specific rules aimed at concealing trial arm allocation from cause of death reviewers, such that any misclassification would be unrelated to trial arm. In addition to UCD assignment, reviewers were requested to guess trial arm allocation, and to provide reasons for their guesses. These reasons were used to revise vignette-writing rules and further standardise information across trial arms, therefore reducing any potential biases in the assignment of UCD arising from the beliefs of reviewers about screening.

## Methods

### Study design

**Figure 1:**
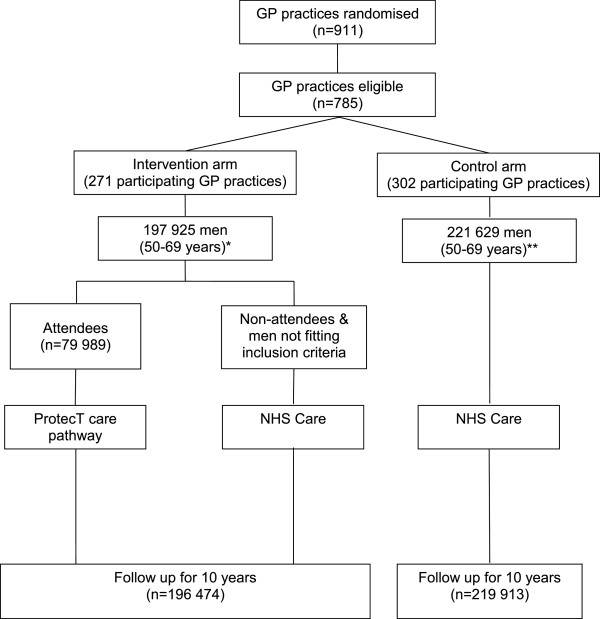
**CAP trial design.** *Further n = 1451 excluded due to prostate cancer pre-randomisation, or failed to trace at HSCIC. **Further n = 1716 men excluded due to prostate cancer pre-randomisation, failed to trace at HSCIC, or refused.

CAP is a randomised-controlled trial (RCT) that evaluates the effectiveness and cost-effectiveness of population-based prostate-specific antigen (PSA) testing for prostate cancer in the UK. The primary outcome of the trial is prostate cancer mortality at ten years (median) follow-up; secondary outcomes are disease stage and grade, progression and all-cause mortality. The trial design (Figure 1) has been reported fully elsewhere [17]. Briefly, between 2001 and 2007 over 550 General Practitioner (GP) practices in eight centres in England and Wales were cluster-randomised to either a single round of PSA testing (intervention) or the National Health Service (NHS) prostate cancer risk management programme [18]. Over 415,000 men aged 50–69 years were included, representing approximately 8% of the male population of England and Wales in this age group. Men in the intervention arm diagnosed with localised prostate cancer through PSA testing were eligible to participate in an embedded randomised-controlled trial - the ProtecT (Prostate testing for cancer and Treatment) trial that evaluates three treatments for clinically localised disease: active monitoring (regular PSA testing and review), radical conformal radiotherapy and radical prostatectomy (ISRCTN20141297) [19]. Approximately 59% of intervention arm men did not respond to the PSA testing invitation (non-attendees) or were not invited for a PSA test because they did not fulfil the Protect inclusion criteria (they were excluded by their GP because of serious co-morbidity or severe mental illness) (Figure 1), and so followed standard NHS prostate cancer risk management. All eligible men in CAP without a pre-randomisation prostate cancer diagnosis were identified and flagged with the regional cancer registries and the Health and Social Care Information Centre (HSCIC) for notification of subsequent cancer diagnoses, embarkations and deaths.

### Clinical vignettes

It was not feasible to review all deaths, which are expected to number over 80,000 during the 10 years of follow-up for the primary outcome. The sub-set of deaths reviewed followed pre-defined criteria adapted from the Prostate, Lung, Colorectal and Ovarian (PLCO) Cancer Screening Trial [1]. These criteria included all men with an incident diagnosis of prostate cancer, regardless of cause of death on the death certificate, and all men with a certified cause of death potentially related to prostate cancer (based on pre-specified ICD 9 or ICD 10 codes in parts 1 or 2 of the death certificate).

Hospital medical records were scrutinised by trained researchers to abstract clinical data onto a standardised proforma, including: symptoms and signs of prostate cancer presence and progression, diagnostic and monitoring tests, histological grade of cancer, tumour stage, treatments received and outcome, complications of prostate cancer and its treatment, and co-morbidities, including other suspected or diagnosed cancers and cardiovascular diseases. The researchers used this information to write a short vignette for each case, arranged in five sections relating to the clinical pathway (clinical features at diagnosis; treatments received; prostate cancer progression; progression of co-morbidities; end of life), and followed by a summary section where a short overview was given (see Additional file 1). All researchers received regular training in trial-specific methodologies, such as data extraction and vignette writing, clinical features of prostate cancer and other common co-morbidities, histology and radiology reporting, as well as the broader aspects of screening trials.

The Cause of Death Evaluation (CODE) Committee is made up of ten external medically qualified reviewers from various clinical specialities (Urology, Oncology, Pathology and Palliative Medicine). A panel of up to four members evaluated each vignette independently and completed a CODE questionnaire, in which they assigned the UCD to one of five categories: definite, probable, possible, unlikely and definitely not prostate cancer deaths, using a pre-defined algorithm adapted from the PLCO and European Randomized Study of Screening for Prostate Cancer (ERSPC) trials [1, 2]. For quality assurance, a random 20% of each researcher’s vignettes were independently reviewed by a urologist for accuracy and completeness using the original medical records, and feedback was given to researchers. Additional information for quality assurance was collected from the CODE questionnaires and required reviewers to subjectively rate the quality of vignettes for adequacy, relevance and clarity, based on a Likert scale of 1–10 (where 1 = poor and 10 = excellent), and to rate their confidence in their UCD decision, based on a Likert scale of 1–5 (where 1 = not at all confident and 5 = extremely confident).

### Masking of trial arm

Researchers and CODE reviewers were blinded to information on the death certificate. Researchers followed specific rules when writing vignettes to mask trial arm allocation and screening status, and sought to standardise clinical information submitted for UCD ascertainment. Initial rules (phase 1) prevented any mention of: i) the ProtecT trial, ‘screening’, or words suggestive of, or specific to, the treatment trial: e.g. ‘3 arm trial’; ‘information appointment’; ‘randomisation’; ‘research nurse’; ’PSA doubling time’; or ‘active monitoring’ (a term used in the ProtecT trial to describe one of three treatment arms; the term ‘conservative treatment’ was to be used instead); ii) participation in other clinical trials, since this would indicate non-participation in the ProtecT trial; and iii) source of original referral (e.g. GP or clinic). In January 2011, in response to a query by the CAP Data Monitoring Committee about the adequacy of reviewers’ blinding, the third rule was tightened so that vignettes did not include any reference to the way in which a man presented or his symptoms at diagnosis.

Several more rules were introduced in June 2011 (phase 2) to further standardise the information presented for UCD assignment: i) age at diagnosis was no longer to be stated in the summary section as men in the intervention arm attending PSA testing as part of ProtecT were aged 50–69 years, whilst those in the control arm could be older at diagnosis; ii) only the PSA test result at diagnosis, and the last three readings or significant PSA measures (e.g. evidence of biochemical failure to treatment) were to be presented to avoid the identification of men allocated to the ‘active monitoring’ treatment arm of ProtecT who received very regular and frequent PSA tests; iii) ‘PSA at diagnosis’ was defined as the reading closest to diagnosis (usually at trans-rectal ultrasound (TRUS)/biopsy), rather than the result at the initial PSA testing clinic (potentially identifying the intervention arm) because some men in the control arm were initially referred without having undergone a PSA test; iv) for investigations at the time of presentation and initial treatments, dates were to be reported in the format of mm/yyyy rather than dd/mm/yyyy. This was because men in the intervention arm diagnosed via PSA testing may have followed a clearly identifiable pathway, obvious by date (PSA, TRUS/biopsy, staging investigations, treatment), whereas in the control arm the sequence of investigations sometimes differed depending on clinical presentation, and in some cases treatment was initiated before all investigations were completed.

Reviewers’ feedback from the CODE questionnaires was used to analyse whether standardised information was being presented across both trial arms. For each assessment, reviewers were asked “what arm of the trial was this man in?” and given three possible options: intervention (invited for PSA testing) arm, control arm or unsure. They were also asked to give reasons for their decision (free text). In this paper we examine the reasons reviewers gave for their choice of trial arm and the extent to which reviewers were able to correctly identify trial arm allocation before (phase 1) and after (phase 2) the revision of vignette writing rules.

### Data analysis

Misclassifications due to a reviewer’s beliefs about screening are unlikely to be differential if those participants the reviewer thinks are in a particular trial arm are actually equally split between screening and control arms. Hence the misclassifications due to beliefs about screening are non-differential between the actual screening and control arms. We can explore this by looking first at those participants the reviewer thinks are in the screening arm and secondly at those participants the reviewer believes are in the control arm, and ascertaining for each in turn if there are equal proportions that are actually in the screening and control arms. By working with percentages of participants who are truly in the screening arm and, separately, who are truly in the control arm, then for participants thought by a reviewer to be in the screening arm we are still looking for equal percentages in the actual screening and control arms to avoid differential misclassification. This is the case even if different numbers of deaths in the two trial arms have been through the review process.

### Ethics approval

CAP has been approved by the Trent Multi-centre Research Ethics Committee (MREC). Reference numbers are: MREC/03/4/093 (12 February 2004) and 05/MRE04/78 (24 November 2005). Approval for flagging of men in the control arm and non-responders in the intervention-arm was obtained from the UK Patient Information Advisory Group (PIAG) (now the Confidentiality Advisory Group (CAG)), under Section 251 of the NHS Act 2006 (reference PIAG 4–09 (k)/2003).

## Results

To January 2014, 1313 independent CODE reviewer assessments of UCD for 605 cases (vignettes) were completed across both study arms. Of these, 1195 assessments (553 cases) included data on the reviewer’s opinion of trial arm (in the earliest assessments reviewers were not questioned about trial arm allocation). There were 96 assessments (44 cases) that were excluded as they related to vignettes written during the 6 month period between January and June 2011 when there had been partial revision of the vignette rules. Of the remaining 1099 assessments, 510 (212 cases) related to vignettes written during phase 1 (prior to January 2011); and 589 assessments (297 cases) related to phase 2 (post-June 2011). The mean quality score assigned to all vignettes by CODE reviewers was the same for both phases (mean: 8.7), as was the reviewer’s confidence in their UCD assessment (mean: 4.5).

### Phase 1

Table 1 shows the reasons reviewers gave for their choice of trial arm in both phases. Because the trial is ongoing, absolute numbers (i.e. of deaths by trial arm) cannot be disclosed so the information is presented here as percentages of the total number in each trial arm. In phase 1, reviewers were unsure of trial arm allocation in 33% of intervention and 30% of control arm assessments. Initially, decisions for the correct identification of intervention arm men were based on the fact that men were asymptomatic at presentation (10%), or because of study-specific criteria that may have suggested the presence of screening, such as the timing of a PSA test result preceding TRUS biopsy, or regular intervals between PSA results (7%). It is important to recognise that since over half of the men allocated to the intervention arm did not accept the invitation for screening or were ineligible for screening and did not have a PSA test (see Figure 1), cancers detected among ‘non-attendees’ would be more akin to control arm cancers. Consequently, intervention arm men were incorrectly assigned to the control arm by reviewers because of the absence of a PSA test (21%) or other study-specific criteria which may have implied the absence of screening (9%), or because men were symptomatic at diagnosis (12%). Correct decisions about the control arm were also based on the absence of either a PSA test result (31%), or prostate cancer diagnosis (8%); or because men were symptomatic or had advanced disease at presentation (13%).

**Table 1:** **Reasons given for choice of trial arm**

		Phase I (N = 510)			Phase 2 (N = 589)		
Correct arm	Reason given	Correct	Incorrect	Unsure	Correct	Incorrect	Unsure
	**1. Factors relating to initial presentation**	**10%**	**14%**	**1%**	**6%**	**24%**	**0%**
	a. Asymptomatic, low PSA test result, localised disease, early presentation	10	2	0	6	3	0
	b. Symptomatic, high PSA test result, locally advanced/metastatic disease, late presentation	0	12	1	0	21	0
	**2. Study-specific factors**	**8%**	**30%**	**4%**	**3%**	**22%**	**2%**
**Intervention**	a. No formal prostate cancer diagnosis	0	3	0	0	2	0
**arm**	b. PSA test not performed	0	21	0	0	11	0
	c. Timing of PSA test, investigations or treatments	4	5	2	1	2	1
	d. Presence of co-morbidities or other cancers	0	0	1	0	4	1
	e. Investigations, treatments received	3	0	0	1	2	0
	f. Other study-specific factors	1	1	1	1	1	0
	**3. No reason stated**	**3%**	**1%**	**25%**	**0%**	**1%**	**42%**
	***(Total for each phase = 100%)***	***21%***	***46%***	***33%***	***9%***	***46%***	***45%***
	**1. Factors relating to initial presentation**	**14%**	**1%**	**1%**	**23%**	**3%**	**3%**
	a. Asymptomatic, low PSA test result, localised disease, early presentation	1	1	1	0	3	1
	b. Symptomatic, high PSA test result, locally advanced/metastatic disease, late presentation	13	0	0	23	0	2
	**2. Study-specific factors**	**50%**	**1%**	**4%**	**20%**	**2%**	**1%**
**Control**	a. No formal prostate cancer diagnosis	8	0	1	1	0	0
**arm**	b. PSA test not performed	31	0	1	10	0	0
	c. Timing of PSA test, investigations or treatments	6	1	1	2	1	0
	d. Presence of co-morbidities or other cancers	1	0	0	3	0	0
	e. Investigations, treatments received	2	0	0	1	1	1
	f. Other study-specific factors	2	0	1	3	0	0
	**3. No reason stated**	**4%**	**0%**	**25%**	**3%**	**0%**	**44%**
	***(Total for each phase = 100%)***	***68%***	***2%***	***30%***	***46%***	***6%***	***48%***

Table 1 shows the reasons reviewers gave for their choice of trial arm, for correct, incorrect and unsure categories; for both phases. Highlighted in bold are the total percentages. Figures have been rounded so may not add up to 100%.

### Phase 2

In phase 2 there was a reduction in the proportion of correct guesses and much greater uncertainty: reviewers were unsure of the trial arm in 45% of all intervention and 48% of control arm assessments. Incorrect assignment of intervention arm men to the control arm was influenced by the absence of a PSA result (11%) or because the cancer had been detected at a much later biological stage than would be expected as a result of detection by screening, for example, advanced disease at presentation or a high PSA level (21%). Study-specific criteria may have implied exclusion from the ProtecT trial (such as previous cancers, multiple co-morbidities and age at diagnosis). The correct identification of control arm men were based on factors such as advanced disease at presentation or a high PSA test result (23%), or the absence of a PSA test (10%).

### Comparison of phases 1 and 2

During phase 1, for those participants thought by reviewers to be in the screening arm, Table 1 shows that more of these participants were actually in the screening arm (21%) than in the control arm (2%). Similarly, for those participants thought to be in the control arm by reviewers, more of these participants were in the control arm (68%) than in the screening arm (46%). Consequently, during Phase 1, misclassifications due to the reviewer’s beliefs about screening could be differential between the two trial arms, and potentially bias estimates of the effect of screening on prostate cancer mortality.

The picture has changed during phase 2, Table 1 showing that for participants thought by reviewers to be in the screening arm, 9% were but 6% were actually in the control arm. For participants thought by reviewers to be in the control arm, there were actually 46% in each of the screening and control arms. Hence in this situation, any misclassifications due to the reviewer’s beliefs about screening are likely to be non-differential across the actual trial arms, and only a moderate underestimate of the effect of screening on prostate cancer mortality will result.

## Discussion

Our analysis of 1099 assessments of 509 vignettes by an endpoint committee of cause of death reviewers showed that even relatively minor details, such as the timing and sequence of investigations or treatments, gave clues about the trial arm to which the man belonged. With the further standardisation of the vignette-writing rules in phase 2, the percentage of cases whereby reviewers were unable to determine trial arm increased. The level of uncertainty for the intervention arm men increased from 33% in phase 1 to 45% in phase 2, while the level of uncertainty for the control arm men increased from 30% in phase 1 to 48% in phase 2.

Importantly, the further standardisation of vignettes would have likely shifted any misclassifications due to reviewers’ belief about screening from differential to non-differential across trial arms. The simple strategy of vignette standardisation equalised and lowered the difference in the proportions of correct and incorrect trial arm allocations (from 21% compared to 2% in phase 1 to 9% compared to 6% in phase 2 for intervention arm guesses; and from 68% compared to 46% in phase 1 to 46% compared to 46% in phase 2 for control arm guesses). Data from phase 2 showed that reviewers were now just as likely to make an intervention arm guess among intervention arm cases (and so guess correctly) as they were to make an intervention arm guess among control arm cases (and so guess incorrectly). Similarly, there was equal likelihood of making a control arm guess among control arm cases (and so guess correctly), as of making a control arm guess among intervention arm cases (and so guess incorrectly). This equalisation should reduce any potential bias due to beliefs that the reviewers may have about the effect of the intervention on outcome (i.e. misclassifications are non-differential across trial arms).

Despite the widely recognised importance of blinding endpoint reviewers in order to reduce bias, blinding methodologies are generally poorly reported [20, 21]. A recent comparison of the cause of death verification process in four screening trials – the Health Insurance Plan of New York breast screening trial (HIP), the Minnesota Colon Cancer Control Study of faecal occult blood testing (MCCCS), and the Johns Hopkins (JHLP) and Mayo Lung Projects (MLP) – found reviewers were given access to all available medical information, including the death certificates [22]. In later trials, more concerted efforts have been taken to conceal trial arm and screening status from reviewers, through the submission of edited copies of the clinical record, with all personal identifiers and references to trial arm or screening status removed [1, 2, 4]. However, such efforts are not always successful, with the treatment assignment (radical prostatectomy versus observation) being correctly guessed for over two-thirds of cases in the PIVOT trial, despite references to trial arm being redacted before notes were sent to the endpoint committee [23]. To achieve accurate adjudications, subtle clues to treatment assignment based on PSA readings and imaging results could not always be removed.

In the CAP trial of population-based PSA testing for prostate cancer, the standardisation of information submitted for UCD assignment across trial arms was optimised through the use of short clinical summaries written by trained researchers in the context of a rigorous quality assurance process, rather than the submission of complete sets of medical notes for review, which may be more difficult to standardise, not only because of the sheer volume of data involved and prohibitive costs of copying and screening records, but also because the language used in hospital correspondence can give subtle clues about screening status, however well-edited. The strength of the CAP trial approach was in enabling researchers to carefully word vignettes to conceal trial arm and screening status, standardise terminology and include the same level of detail across trial arms, while providing sufficient information for accurate determination of UCD. In fact, even after the refinement of vignette writing rules, the quality of vignettes was not compromised. The mean quality score assigned to all vignettes by CODE reviewers was the same for both phase 1 and phase 2 (mean: 8.7). Though some clinical data were omitted from vignettes in order to achieve standardisation of information, this did not impact on the confidence of reviewers in assigning UCD; the mean rating given by the reviewers for their confidence in their UCD decision was 4.5 out of 5 in both phases (where 1 = not at all confident and 5 = extremely confident). In addition, implementing such vignette-based blinding procedures sped up the vignette writing process (rather than demanding more time), as the amount of clinical information presented became more streamlined with standardisation.

In this study, our aim was not to measure blinding effectiveness, as such, using formal tests of blinding. In fact in CONSORT 2010, mention of how the success of blinding might be evaluated was specifically removed, in view of the interpretational and measurement difficulties [24]. In our study, we aimed to improve blinding qualitatively by reducing systematic error, rather than attempting to measure blinding success quantitatively, as we recognise that it is difficult for such measurement to yield meaningful interpretative data.

Our analysis raises two issues. First, the accurate assignment of UCD requires a careful balance to be struck between the amount and type of clinical information presented and the adequacy of blinding achieved. Cancer screening trials face inherent difficulties in this context, since the rationale for population-based screening is the detection of early cancers before they present clinically, when potentially curative treatments are available [25]. Consequently in our analysis, reviewers were influenced by low PSA test results or early or localised disease in correctly identifying intervention arm men; whereas high PSA test results or advanced disease at diagnosis were frequently used as a basis for identifying control arm men correctly. In cancer screening, other scenarios involving the incidental diagnosis of the target cancer also pose a challenge for masking trial arm without compromising UCD ascertainment: for example, where a prostate cancer is diagnosed incidentally following a radical cystoprostatectomy for bladder cancer, the inclusion of histological details might compromise blinding, but the grade and stage of bladder cancer would be needed to ensure accurate UCD assignment.

Secondly, as in many other studies, CAP uses the endpoint committee for final UCD assignment but have no means of ascertaining if any misclassifications have occurred at this level. Therefore it is not possible to determine directly whether there are differential or non-differential misclassifications. However, the results presented in this report clearly demonstrate that vignette standardisation moved the general trend from potential differential to non-differential misclassifications, if any of this should occur.

## Conclusions

In CAP, researcher-written vignettes were used to mask external cause of death reviewers to the allocation of trial arm in order to reduce any potential biases in the verification of UCD. Feedback from the reviewers was used to standardise and streamline the information presented to the endpoint committee for UCD assignment. This has been shown to improve masking of trial arm without compromising vignette quality. This finding is particularly relevant to RCTs where the primary outcome is cause-specific mortality determined by independent cause of death review.

## Electronic supplementary material

Additional file 1: **Vignette for cause of death review.** (PDF 79 KB)

## Abbreviations

CAP: Cluster randomised triAl of PSA testing for Prostate cancer
CODE: Cause of death evaluation committee
DMC: Data monitoring committee
ERSPC: European Randomized Study of Screening for Prostate Cancer
GP: General practitioner
HSCIC: Health and Social Care Information Centre
HIP: The Health Insurance Plan
ICD: International classification of diseases
JHLP: Johns Hopkins Lung Project
MLP: Mayo Lung Project
MCCCS: Minnesota Colon Cancer Control Study
NHS: National Health Service
PLCO: Prostate, Lung, Colorectal, Ovarian screening trial
ProtecT: Prostate testing for cancer and Treatment
PSA: Prostate-specific antigen
TRUS: Trans-rectal ultrasound
UCD: Underlying cause of death.
