# Supplementary material for: Standardisation of information submitted to an endpoint committee for cause of death assignment in a cancer screening trial – lessons learnt from CAP (Cluster randomised triAl of PSA testing for Prostate cancer)
Source: BMC Med Res Methodol. 2015 Jan 23;15:6. doi: 10.1186/1471-2288-15-6 (PMC4429825; doi:10.1186/1471-2288-15-6)
Supplement: Supplementary file 1 — Additional file 1: Vignette for cause of death review. (PDF 79 KB) [file 12874_2014_1176_MOESM1_ESM.pdf]

Vignette for cause of death review

* Categories affected by changes in vignette writing rules (see text)

Study ID<br>Number<br><br>Initials<br><br>Date of<br>birth	Date of diagnosis<br>(of prostate cancer)<br><br>Date of death<br><br>Age at death

**Clinical features at diagnosis**	
Symptoms at diagnosis (comment on presence of bony pain, weight loss, cachexia, loss of appetite, obstructive uraemia)*	
Gleason Score at diagnosis (with dates)	
Clinical stage (TNM)	
Pathological stage (TNM)	
Co-morbidities with dates of diagnosis	
Other primary cancers with dates of diagnosis	
PSA level at diagnosis with dates*	
Radiological evidence of local spread at diagnosis	
Radiological evidence of metastases at diagnosis	
**Treatments received**	
Initial treatments (dates)	
Hormone therapy (start date)	
Maximum androgen blockade (start date)	
Orchidectomy (date)	
Chemotherapy (start date)	
Treatment for complications of treating prostate cancer with dates (if available)	
**Prostate cancer progression**	
Serial PSA levels (dates) – last 3 PSA measurements*	

Serum testosterone	
Radiological evidence of metastases	
Other indications or complications of disease progression	
Date of recurrence following radical surgery or radiotherapy	
Palliative care referrals and treatments	
**Progression of co-morbidities**	
Treatment/ admission for co-morbidity with dates (if available)	
**End of life**	
Symptoms in last 3-6 months (i.e. bone pain, weight loss, cachexia, loss of appetite, obstructive uraemia)	
Last consultation: speciality & date	
Was a DS1500 report issued?	
**Post mortem findings**	

Summary: Especially note any progression of prostate cancer or progression of co-morbidities, or any factors that may contribute to the cause of death.
